# Complete genome sequence of the marine methyl-halide oxidizing *Leisingera methylohalidivorans* type strain (DSM 14336^T^), a representative of the *Roseobacter* clade

**DOI:** 10.4056/sigs.4297965

**Published:** 2013-10-04

**Authors:** Nora Buddruhs, Olga Chertkov, Jörn Petersen, Anne Fiebig, Amy Chen, Amrita Pati, Natalia Ivanova, Alla Lapidus, Lynne A. Goodwin, Patrick Chain, John C. Detter, Sabine Gronow, Nikos C. Kyrpides, Tanja Woyke, Markus Göker, Thorsten Brinkhoff, Hans-Peter Klenk

**Affiliations:** 1 Leibniz-Institute DSMZ – German Collection of Microorganisms and Cell Cultures, Braunschweig, Germany; 2 Los Alamos National Laboratory, Bioscience Division, Los Alamos, New Mexico, USA; 3 Biological Data Management and Technology Center, Lawrence Berkeley National Laboratory, Berkeley, California, USA; 4 DOE Joint Genome Institute, Walnut Creek, California, USA; 5 Theodosius Dobzhansky Center for Genome Bioinformatics, St. Petersburg State University, St. Petersburg, Russia; 6 Algorithmic Biology Lab, St. Petersburg Academic University, St.Petersburg, Russia; 7 Institute for Chemistry and Biology of the Marine Environment (ICMB), Oldenburg, Germany

**Keywords:** Methylotrophy, methyl halides, extrachromosomal elements, *Alphaproteobacteria*, *Rhodobacteraceae*, *Roseobacter* clade, aerobe

## Abstract

*Leisingera methylohalidivorans* Schaefer *et al*. 2002 emend. Vandecandelaere *et al*. 2008 is the type species of the genus *Leisingera*. The genus belongs to the *Roseobacter* clade (*Rhodobacteraceae*, *Alphaproteobacteria*), a widely distributed lineage in marine environments. *Leisingera* and particularly *L. methylohalidivorans* strain MB2^T^ is of special interest due to its methylotrophy. Here we describe the complete genome sequence and annotation of this bacterium together with previously unreported aspects of its phenotype. The 4,650,996 bp long genome with its 4,515 protein-coding and 81 RNA genes consists of three replicons, a single chromosome and two extrachromosomal elements with sizes of 221 kb and 285 kb.

## Introduction

Strain MB2^T^ (= DSM 14336^T^ = ATCC BAA-92^T^) is the type strain of the species *L. methylohalidivorans*. *L. methylohalidivorans* MB2^T^ was isolated from a tide pool off the coast of California and first described in 2002 by Schaefer *et al*. [1]. The species was emended by Martens *et al*. [2] and by Vandecandelaere *et al.* [3].

*L. methylohalidivorans* [1] is the type species of the genus *Leisingera*, which currently contains two more validly named species, *L. aquimarina* [3] and *L. nanhaiensis* [4]. The genus belongs to the *Roseobacter* clade, a widely distributed lineage in marine habitats with considerable metabolic versatility [5-8]. The genus name is derived in honor of Thomas Leisinger, on the occasion of his retirement and for his contributions to the understanding of the biochemistry of bacterial methyl halide metabolism. *Leisingera* comprises organisms associated with their ability to grow by oxidation of methyl groups of methionine and, at least for *L. methylohalidivorans,* by oxidation of methyl halides as a sole energy and carbon source [1]. Methyl halide-degrading bacteria potentially play an important role in mitigating ozone depletion resulting from methyl chloride and methyl bromide emissions [9].

Here we present a summary classification and a set of features for *L. methylohalidivorans* MB2^T^, including novel aspects of its phenotype, together with the description of the complete genomic sequencing and annotation.

## Classification and features

### 16S rRNA analysis

**Figure 1:**
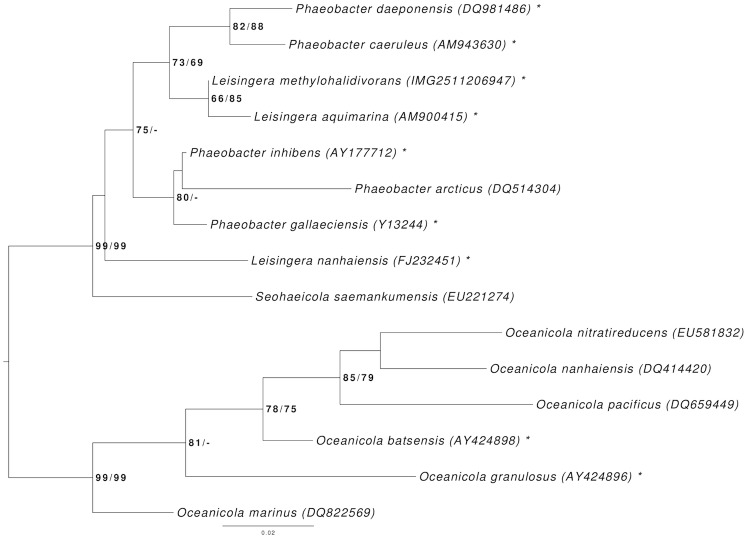
Phylogenetic tree highlighting the position of *L. methylohalidivorans* relative to the type strains of the other species within the genus *Leisingera* and the neighboring genera *Phaeobacter* and *Oceanicola*. The tree was inferred from 1,385 aligned characters of the 16S rRNA gene sequence under the maximum likelihood (ML) criterion as previously described [10]. *Oceanicola* were included in the dataset for use as outgroup taxa. The branches are scaled in terms of the expected number of substitutions per site. Numbers adjacent to the branches are support values from 1,000 ML bootstrap replicates (left) and from 1,000 maximum-parsimony bootstrap replicates (right) if larger than 60% [10]. Lineages with type strain genome sequencing projects registered in GOLD [11] are labeled with one asterisk, those also listed as standard 'Complete and Published' with two asterisks [12-14]. The genomes of three more *Leisingera* and *Phaeobacter* species are published in the current issue of *Standards in Genomic Science* [15-17].

Figure 1 shows the phylogenetic neighborhood of *L. methylohalidivorans* DSM 14336^T^ in a 16S rRNA based tree. The sequences of the five 16S rRNA gene copies in the genome differ from each other by up to two nucleotides, and differ by up to four nucleotides from the previously published 16S rRNA sequence (AY005463) [1].

A representative genomic 16S rRNA sequence of *L. methylohalidivorans* DSM 14336^T^ was compared with the Greengenes database for determining the weighted relative frequencies of taxa and (truncated) keywords as previously described [10]. The most frequently occurring genera were *Ruegeria* (32.5%), *Phaeobacter* (28.2%), *Roseobacter* (14.2%), *Silicibacter* (12.9%) and *Nautella* (3.5%) (143 hits in total). Regarding the three hits to sequences from the species, the average identity within HSPs was 99.9%, whereas the average coverage by HSPs was 99.1%. Regarding the single hit to sequences from other species of the genus, the average identity within HSPs was 99.4%, whereas the average coverage by HSPs was 99.8%. Among all other species, the one yielding the highest score was *'Leisingera aquamarina'* (AM900415; a misnomer for *L. aquimarina*) [3], which corresponded to an identity of 99.4% and an HSP coverage of 99.8%. (Note that the Greengenes database uses the INSDC (= EMBL/NCBI/DDBJ) annotation, which is not an authoritative source for nomenclature or classification.) The highest-scoring environmental sequence was AY007684 ('marine isolate JP88.1'), which showed an identity of 98.1% and an HSP coverage of 100.1%. The most frequently occurring keywords within the labels of all environmental samples which yielded hits were 'microbi' (4.1%), 'marin' (2.8%), 'structur' (2.3%), 'biofilm' (2.1%) and 'swro' (2.1%) (100 hits in total). Environmental samples which yielded hits of a higher score than the highest scoring species were not found. This indicates that the species is rarely detected in the environment.

### Morphology and physiology

**Figure 2:**
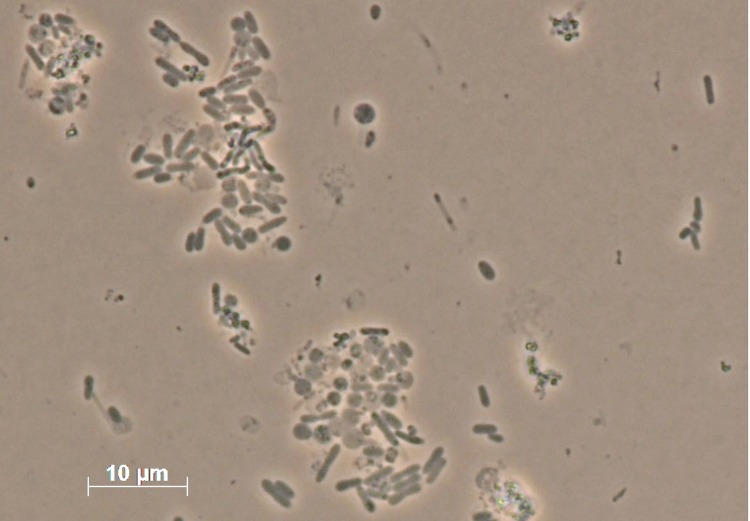
Optical micrograph of *L. methylohalidivorans* MB2^T^

The characteristics of strain MB2^T^ are summarized in Table 1. Cells of *L. methylohalidivorans* MB2^T^ are Gram-negative and motile, obligatory aerobic and rod-shaped or rather pleomorphic, depending on the cultivation medium (Table 1) [1]. Colonies are non-pigmented, smooth, with an entire edge when grown on solid media regardless of the carbon source [1]. The strain forms single or paired rods (1.1–1.4 x 0.4–0.5 µm) when grown with methyl halides, methionine or DMS on mineral medium. When cultured with yeast extract or glycine betaine, the rods become enlarged and elongated (2.4–8.2 x 0.7–0.8 µm). Yeast-grown cell lines returned to mineral salts medium with MeBr as the substrate reestablish their original form [1]. Cells grown on marine broth showed the standard ovoid rod morphology (Figure 2).

**Table 1:** Classification and general features of *L. methylohalidivorans* MB2^T^ according to the MIGS recommendations [18] published by the Genome Standards Consortium [19].

**MIGS ID**	**Property**	**Term**	**Evidence code**
	Current classification	Domain *Bacteria*	TAS [20]
		Phylum *Proteobacteria*	TAS [21]
		Class *Alphaproteobacteria*	TAS [22,23]
		Order *Rhodobacterales*	TAS [23,24]
		Family *Rhodobacteraceae*	TAS [23,25]
		Genus *Leisingera*	TAS [1-3]
		Species *Leisingera methylohalidivorans*	TAS [1,3]
MIGS-7	Subspecific genetic lineage (strain)	MB2^T^	TAS [1]
MIGS-12	Reference for biomaterial	Schaefer *et al.* 2002	TAS [1]
	Gram stain	negative	TAS [1]
	Cell shape	ovoid rods/ pleomorphism	TAS [1]
	Motility	motile	TAS [1]
	Sporulation	non-sporulating	TAS [1]
	Temperature range	mesophile	TAS [1]
	Optimum temperature	27°C	TAS [1]
	Salinity	halophile	TAS [1]
MIGS-22	Relationship to oxygen	obligatory aerobic	TAS [1]
	Carbon source	complex substrates, methyl halides, DMS, methionine, glycine betaine	TAS [1]
MIGS-6	Habitat	aquatic, sea water	TAS [1]
MIGS-6.2	pH	7.7	TAS [1]
MIGS-15	Biotic relationship	free-living	TAS [1]
MIGS-14	Known pathogenicity	none	TAS [1]
	Biosafety level	1	TAS [26]
MIGS-23.1	Isolation	tide pool	TAS [1]
MIGS-4	Geographic location	USA, Washington DC	TAS [1]
MIGS-5	Time of sample collection	2002 or before	TAS [1]
MIGS-4.1	Latitude	38.90	TAS [1]
MIGS-4.2	Longitude	-77.03	TAS [1]

Evidence codes - IDA: Inferred from Direct Assay; TAS: Traceable Author Statement (i.e., a direct report exists in the literature); NAS: Non-traceable Author Statement (i.e., not directly observed for the living, isolated sample, but based on a generally accepted property for the species, or anecdotal evidence). Evidence codes are from the Gene Ontology project [27].

Growth also occurs on casamino acids and weakly on TSA. No growth was observed on NA, R2A, PYG, carbon sources, amino acids (other than methionine) and small organic acids. Cells are catalase- and oxidase-positive. The strain does not hydrolyze starch, aesculin or gelatin, and tested positive for leucine arylamidase activity; weak valine arylamidase and naphtol-AS-BI-phosphohydrolase activities. No activity is detected for alkaline phosphatase esterase (C4), esterase lipase (C8), lipase (C14), cystine arylamidase, trypsin, α-chymotrypsin, acid phosphatase, α-galactosidase, β-galactosidase, β-glucuronidase, α-glucosidase, *N*-acetyl-β-glucosaminidase, α-mannosidase, α-fucosidase, arginine dihydrolase or urease. It is unable to use nitrate as an electron acceptor. Vitamins are not necessary for growth. Strain MB2^T^ does not degrade tyrosine, casein or DNA. No indole production or fermentation of glucose were detected [1,3]. As a marine bacterium isolated from seawater, growth occurred over a salinity range of 10–60 g/L NaCl , with an optimum at the salinity of seawater. The optimum Mg^2+^ concentration for strain MB2^T^ was 40–80 mM, which overlaps with the 54 mM concentration found in seawater [1].

Strain MB2^T^ is susceptible to penicillin G (50 µg), cefoxitin (30 µg), erythromycin (15 µg), streptomycin (25 µg) and tetracycline (30 µg). It is moderately susceptible to gentamicin (10 µg) but resistant to vancomycin (30 µg), trimethoprim (1.25 µg) and clindamycin (2 µg) [1,3].

The utilization of carbon compounds by *L. methylohalidivorans* DSM 14336^T^ was also determined for this study using Generation-III microplates in an OmniLog phenotyping device (BIOLOG Inc., Hayward, CA, USA). The microplates were inoculated at 28°C with a cell suspension at a cell density of 95-96% turbidity and dye IF-A. Further additives were vitamin, micronutrient and sea salt solutions. The exported measurement data were further analyzed with the opm package for R [28,29], using its functionality for statistically estimating parameters from the respiration curves such as the maximum height, and automatically translating these values into negative, ambiguous, and positive reactions. The strain was studied in two independent biological replicates, and reactions with a different behavior between the two repetitions were regarded as ambiguous and are not listed below.

The strain gave positive reactions for 1% NaCl, 4% NaCl, D-glucose, D-mannitol, D-glucose-6-phosphate, D-aspartic acid, L-alanine, L-arginine, L-glutamic acid, L-histidine, L-pyroglutamic acid, L-serine, D-galacturonic acid, glucuronamide, quinic acid, D-saccharic acid, D-lactic acid methyl ester, α-keto-glutaric acid, L-malic acid, nalidixic acid, acetoacetic acid, propionic acid and acetic acid.

The strain was negative for sucrose, pH 6, pH 5, D-melibiose, D-salicin, N-acetyl-D-glucosamine, N-acetyl-D-galactosamine, N-acetyl-neuraminic acid, 8% NaCl, D-galactose, 3-O-methyl-D-glucose, D-fucose, L-fucose, inosine, 1% sodium lactate, fusidic acid, D-serine, D-sorbitol, D-arabitol, D-fructose-6-phosphate, D-serine, troleandomycin, rifamycin SV, minocycline, lincomycin, guanidine hydrochloride, niaproof 4, pectin, L-galactonic acid-γ-lactone, mucic acid, vancomycin, tetrazolium violet, tetrazolium blue, p-hydroxy-phenylacetic acid, methyl pyruvate, citric acid, bromo-succinic acid, potassium tellurite, α-hydroxy-butyric acid, β-hydroxy-butyric acid, α-keto-butyric acid, sodium formate, aztreonam, butyric acid and sodium bromate.

Regarding the common subset of growth experiments and OmniLog experiments, the results were identical with few exceptions. Expectedly [30], on some substrates respiration was detected by phenotype microarray analysis even though these substrates did not sustain growth.

### Chemotaxonomy

The major respiratory lipoquinone present is Q10 [1]. The polar lipids comprise phosphatidylglycerol, phosphatidylethanolamine, an unidentified phospholipid, two unidentified lipids and an aminolipid. Phosphatidylcholine is not present. The fatty acids comprise C_10:0 3-OH_, C_14:1_, C_12:0 3-OH_, C_16:0_, C_16:0 2-OH_, C_18:1ω9c_, C_18:1ω7c_, C_18:0_ and 11-methyl C_18:1ω7c_. The C_10:0 3-OH_ and C_16:0 3-OH_ fatty acids are ester-linked, while the C_12:0 3-OH_ fatty acid is amide-linked [2].

## Genome sequencing and annotation

### Genome project history

This organism was selected for sequencing on the basis of the DOE Joint Genome Institute Community Sequencing Program (CSP) 2010, CSP 441 “Whole genome type strain sequences of the genera *Phaeobacter* and *Leisingera* – a monophyletic group of physiological highly diverse organisms”. The genome project is deposited in the Genomes On Line Database [11] and the complete genome sequence is deposited in GenBank and the Integrated Microbial Genomes database (IMG) [31]. Sequencing, finishing and annotation were performed by the DOE Joint Genome Institute (JGI) using state of the art sequencing technology [32]. A summary of the project information is shown in Table 2.

**Table 2:** Genome sequencing project information

MIGS ID	Property	Term
MIGS-31	Finishing quality	Finished
MIGS-28	Libraries used	Two Illumina paired-end libraries (270 bp and 9 kb insert size)
MIGS-29	Sequencing platforms	Illumina GAii
MIGS-31.2	Sequencing coverage	382.5 × Illumina
MIGS-30	Assemblers	Allpaths, Velvet 1.1.05, phrap version SPS - 4.24
MIGS-32	Gene calling method	Prodigal 1.4, GenePRIMP
	INSDC ID	INSDC ID CP006773 (cMeth_4145), CP006774 (pMeth_B221), CP006775 (pMeth_A285)
	GenBank Date of Release	September 30, 2013
	GOLD ID	Gi10858
	NCBI project ID	PRJNA74371
	Database: IMG	2512564009
MIGS-13	Source material identifier	DSM 14336
	Project relevance	Tree of Life, carbon cycle, sulfur cycle, environmental

### Growth conditions and DNA isolation

A culture of DSM 14336^T^ was grown aerobically in DSMZ medium 514 [33] at 20°C. Genomic DNA was isolated using a Jetflex Genomic DNA Purification Kit (GENOMED 600100) following the standard protocol provided by the manufacturer but modified by an incubation time of 40 min, the incubation on ice over night on a shaker, the use of additional 10 µl proteinase K, and the addition of 100 µl protein precipitation buffer. DNA is available from DSMZ through the DNA Bank Network [34].

### Genome sequencing and assembly

The draft genome sequence was generated using Illumina sequencing technology. For this genome, we constructed and sequenced an Illumina short-insert paired-end library with an average insert size of 270 bp, which generated 10,989,662 reads. In addition, an Illumina long-insert paired-end library with an average insert size of 9,000 bp was constructed, generating 1,005,012 reads for a total of 1,798 Mb of Illumina data (Feng Chen, unpublished). All general aspects of library construction and sequencing performed can be found at the JGI web site [35]. The initial draft assembly contained 16 contigs in 6 scaffold(s). The initial draft data was assembled with Allpaths [36] and the consensus was computationally shredded into 10 kbp overlapping fake reads (shreds). The Illumina draft data was also assembled with Velvet [37], and the consensus sequences were computationally shredded into 1.5 kbp overlapping fake reads (shreds). The Illumina draft data was assembled again with Velvet using the shreds from the first Velvet assembly to guide the next assembly. The consensus from the second Velvet assembly was shredded into 1.5 kbp overlapping fake reads. The fake reads from the Allpaths assembly and both Velvet assemblies and a subset of the Illumina CLIP paired-end reads were assembled using parallel phrap (High Performance Software, LLC) [38]. Possible mis-assemblies were corrected with manual editing in Consed [38]. Gap closure was accomplished using repeat resolution software (Wei Gu, unpublished), and sequencing of bridging PCR fragments with Sanger technologies. A total of 15 additional sequencing reactions were completed to close gaps and to raise the quality of the final sequence. The total size of the genome is 4,630,996 bp and the final assembly is based on 1,798 Mb of Illumina draft data, which provides an average 382.5 × coverage of the genome.

### Genome annotation

Genes were identified using Prodigal [39] as part of the DOE-JGI genome annotation pipeline [40], followed by a round of manual curation using the JGI GenePRIMP pipeline [41]. The predicted CDSs were translated and used to search the National Center for Biotechnology Information (NCBI) nonredundant database, UniProt, TIGR-Fam, Pfam, PRIAM, KEGG, COG, and InterPro databases. Additional gene prediction analysis and functional annotation was performed within the Integrated Microbial Genomes - Expert Review (IMG-ER) platform [31].

## Genome properties

**Figure 3:**
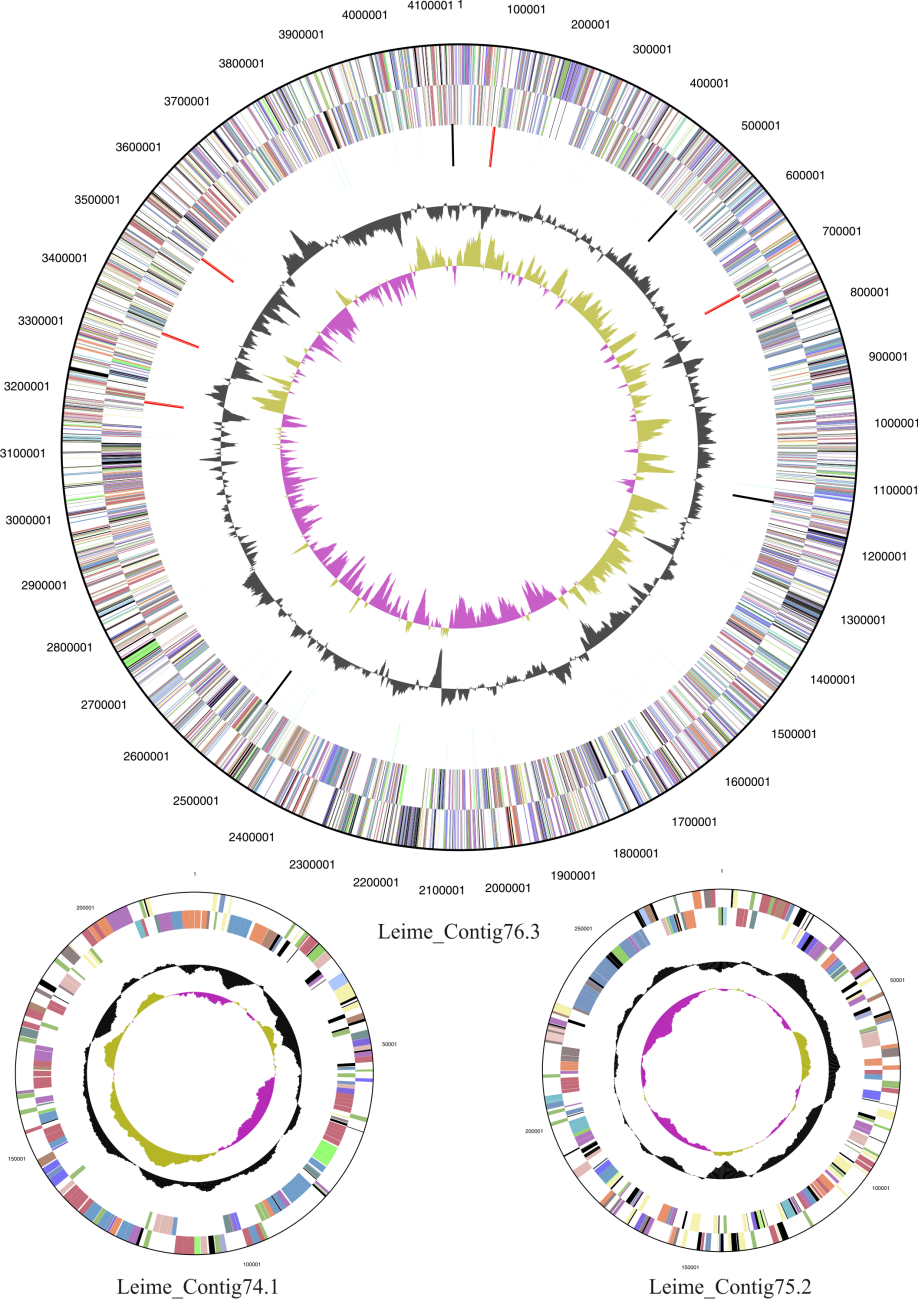
Graphical map of the chromosome (cMeth_4145 = Leime_Contig76.3) and the two extrachromosomal elements (pMeth_B221 = Leime_Contig74.1 and pMeth_A285 = Leime_Contig 75.2).

The *L. methylohalidivorans* DSM 14336^T^ genome statistics are provided in Table 3 and Figure 3. The genome consists of three circular replicons with a total length of 4,650,996 bp and a G+C content of 62.3%. The replicons correspond to a single chromosome (4,144,900 bp in length) and two extrachromosomal elements 220,701 bp and 285,395 bp in length. Of the 4,596 genes predicted, 4,515 were protein-coding genes, and 81 RNAs. In addition, 293 pseudogenes were also identified. The majority of the protein-coding genes (77.4%) were assigned a putative function while the remaining ones were annotated as hypothetical proteins. The distribution of genes into COGs functional categories is presented in Table 4.

**Table 3:** Genome statistics

**Attribute**	**Number**	**% of Total**
Genome size (bp)	4,650,996	100.00
DNA coding region (bp)	3,929,972	84.50
DNA G+C content (bp)	2,898,874	62.33
Number of replicons	3	
Extrachromosomal elements	2	
Total genes	4,596	100.00
RNA genes	81	1.76
rRNA operons	5	
tRNA genes	62	1.35
Protein-coding genes	4,515	98.24
Pseudo genes	293	6.38
Genes with function prediction	3,558	77.42
Genes in paralog clusters	1,675	36.44
Genes assigned to COGs	3,493	76.00
Genes assigned Pfam domains	3,567	77.61
Genes with signal peptides	1,470	31.98
Genes with transmembrane helices	867	18.86
CRISPR repeats	0	

**Table 4:** Number of genes associated with the general COG functional categories

Code	Value	%age	Description
J	179	4.67	Translation, ribosomal structure and biogenesis
A	0	0.00	RNA processing and modification
K	322	8.39	Transcription
L	232	6.05	Replication, recombination and repair
B	3	0.08	Chromatin structure and dynamics
D	34	0.89	Cell cycle control, cell division, chromosome partitioning
Y	0	0.00	Nuclear structure
V	40	1.04	Defense mechanisms
T	161	4.2	Signal transduction mechanisms
M	181	4.72	Cell wall/membrane/envelope biogenesis
N	51	1.33	Cell motility
Z	1	0.03	Cytoskeleton
W	0	0.00	Extracellular structures
U	62	1.62	Intracellular trafficking, secretion, and vesicular transport
O	138	3.60	Posttranslational modification, protein turnover, chaperones
C	237	6.18	Energy production and conversion
G	154	4.01	Carbohydrate transport and metabolism
E	459	11.96	Amino acid transport and metabolism
F	102	2.66	Nucleotide transport and metabolism
H	184	4.8	Coenzyme transport and metabolism
I	150	3.91	Lipid transport and metabolism
P	182	4.74	Inorganic ion transport and metabolism
Q	123	3.21	Secondary metabolites biosynthesis, transport and catabolism
R	465	12.12	General function prediction only
S	377	9.83	Function unknown
-	1,110	24.09	Not in COGs

## Insights into the genome

Genome sequencing of *L. methylohalidivorans* DSM 14336^T^ reveals the presence of two plasmids with sizes of 221 kb and 285 kb (Table 5). The circular conformation of the chromosome and the two extrachromosomal elements have been experimentally validated. The two plasmids contain characteristic replication modules of the DnaA-like and RepABC-type comprising a replicase as well as the *parAB* partitioning operon [42]. The respective replicases that mediate the initiation of replication are designated according to the established plasmid classification scheme [43]. The different numbering of the replicase RepC-8 from the RepABC-type plasmids corresponds to specific plasmid compatibility groups that are required for a stable coexistence of the replicons within the same cell [44].

**Table 5:** General genomic features of the chromosome and extrachromosomal replicons from *L. methylohalidivorans* strain DSM 14336^T^.

**Replicon**	**Scaffold**	**Replicase**	**Length** (bp)	**GC** (%)	**Topology**	**No. Genes^†^**
cMeth_4145	1	DnaA	4,144,900	62	circular	4,135
pMeth_A285	2	DnaA-like I	285,395	62	circular	269
pMeth_B221	3	RepC-8	220,701	62	circular	204

^†^deduced from automatic annotation.

The locus tags of all replicases, plasmid stability modules and the large *virB4* and *virD4* genes of the type IV secretion systems are presented in Table 6. The larger plasmid, pMeth_A285, harbors a postsegregational killing system (PSK) consisting of a typical operon with two small genes encoding a stable toxin and an unstable antitoxin [45]. The smaller plasmid pMeth_B221 contains the *virD2* and *virD4* genes of the type IV secretion system, but it is probably non-conjugative, since the *virB* operon for the formation of a transmembrane channel is missing [46,47].

**Table 6:** Integrated Microbial Genome (IMG) locus tags of *L. methylohalidivorans* DSM 14336^T^ genes^†^

Replicon	Replication initiation		Plasmid stability		Type IV secretion	
	Replicase	Locus Tag	Toxin	Antitoxin	VirB4	VirD4
						
cMeth_4145	DnaA	Meth_0476	-	-	-	-
pMeth_A285	DnaA-like I	Meth_0245	Meth_0528	Meth_0529	-	-
pMeth_B221	RepC-8	Meth_0107	-	-	-	Meth_0035^1^

^†^Genes for the initiation of replication, toxin/antitoxin modules and type IV secretion systems (T4SS) that are required for conjugation.

^1^Presence of adjacent DNA relaxase VirD2.

The 285 kb DnaA-like I replicon pMeth_A285 contains a large type VI secretion system (T6SS) with a size of about 30 kb. The role of this export system was first described in the context of bacterial pathogenesis, but recent findings indicate a more general physiological role in defense against eukaryotic cells and other bacteria in the environment [48]. Homologous T6S systems are present on the DnaA-like I plasmids of *L. aquimarina* DSM 24565^T^ (pAqui_F126) and *Phaeobacter caeruleus* DSM 24564^T^ (pCaer_C109) as well as the RepC-8 type plasmid of *Phaeobacter daeponensis* DSM 23529^T^ (pDaep_A276) [12]. This extrachromosomal replicon also harbors a TonB-dependent siderophore receptor (Meth_0471) and genes of a putative ABC-type Fe^3+^ siderophore transport system (Meth_0472 to Meth_0467).

The 221 kb RepC-8 type replicon pMeth_B221 contains five ABC-transporters. One of them, which probably transports nitrate/sulfonate or bicarbonate (Meth_0002, Meth_0001, Meth_0204, Meth_0203), is located adjacent to the large and small subunit genes of the nitrate reductase (EC 1.7.1.4; Meth_0202, Meth_0201) and an anaerobic dehydrogenase (EC 1.7.99.4; Meth_0200) hence indicating a functional role of the plasmid in anaerobic metabolism.

**Figure 4:**
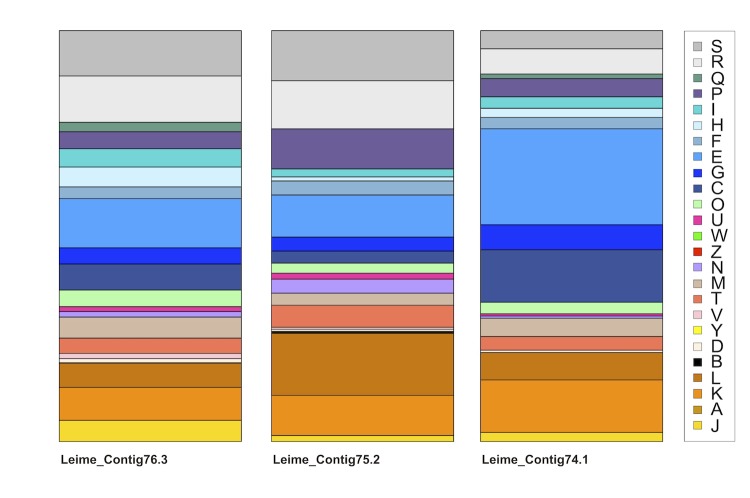
Bar plot of the relative amounts of the COG categories of the chromosome (cMeth_4145 = Leime_Contig76.3, left) and both extrachromosomal elements (pMeth_A285 = Leime_Contig 75.2, center, and pMeth_ B221 = Leime_Contig74.1, right). The COG functional categories are described in Table 4.

To quantify the differences in COG functional categories between the three replicons and to determine the over-represented categories, we used approaches based on entropy and the broken-stick distribution, respectively. We applied these methods to all genes that were assigned to a COGs category from either genome [49]. Figure 4 shows the bar plot of the COG categories of the replicons [46]. The analysis revealed one over-represented COG category for the small extrachromosomal element (pMeth_B221), i.e. “amino acid metabolism” (category E). For instance, this replicon encodes nine spermidine/putrescine transporter sequences (Meth_0060, _0061, _0062, _0063, _0133, _0134, _0135, _0136, _0169) suggesting that these compounds are an important source for *L. methylohalidivorans* DSM 14336^T^. Spermidine and putrescine are produced in marine phytoplankton and zooplankton to regulate cell proliferation and bloom formation [50].

The COG category P (“inorganic ion transport and metabolism”) (Figure 4) is highly represented in the larger extrachromosomal element (Meth_0238, _0261, _0263, _0264, _0265, _0266, _0303, _0305, _0355, _0360, _0378, _0413, _0414, _0415, _0463, _0468, _0469, _0470, _0471). This replicon encodes a broad spectrum of inorganic transport and regulation systems for sulfate, phosphate, 2-aminoethylphosphate, manganese(II), zinc(II), ferric, ferrous, ferric-citrate, formate, nitrite, calcium(II), sodium, molybdenum and copper.

In accordance with the known ability of *L. methylohalidivorans* DSM 14336^T^ to grow by oxidation of methyl halides [1], the genome analysis revealed the genes for the proposed pathway of methyl chloride metabolism as described by McDonald *et al.* 2002 [9]. Using the JGI-IMG BLASTp tool [51,52], the gene for first methyltransferase I (*cmuA*) indeed yielded a hit to the gene *cmuA* (“predicted cobalamin binding protein”, Meth_2531) in the genome of *L. methylohalidivorans* DSM 14336^T^, with a sequence similarity of 31%. Searching for the second enzyme methyltransferase II (*cmuB*) yielded a hit to the enzyme adjacent to the predicted cobalamin binding protein (“methionine synthase I (cobalamin-dependent), methyltransferase domain”, Meth_2528). For the next enzymes in the methyl-chloride metabolism, we compared the genes *metF*, *folD*, *purU* and *FDH* and found the following results: 39% similarity to a 5,10-methylenetetrahydrofolate reductase (Meth_1763), for *metF*; 56% to a 5,10-methylene-tetrahydrofolate dehydrogenase/Methenyl-tetrahydrofolate cyclohydrolase (Meth_4077, Meth_3180) for *folD*; 36% to a phosphoribosylglycinamide formyltransferase, formyltetrahydrofolate-dependent (Meth_2536) for *purU*; and 79% to a formate dehydrogenase (Meth_4011) for *FDH*.

An estimate of the DNA-DNA hybridization (DDH) similarity between *L. methylohalidivorans* DSM 14336^T^ and the draft genomes of *L. aquimarina* DSM 24565^T^, *L. nanhaiensis* DSM 24252^T^, *P. arcticus* DSM 23566^T^, *P. caeruleus* DSM 24564^T^, *P. daeponensis* DSM 23529^T^, *P. gallaeciensis* CIP 105210^T^ and *P. inhibens* DSM 16374^T^ was generated with the GGDC Genome-to-Genome Distance Calculator version 2.0 [53-55]. This system calculates the distances by comparing the genomes to obtain HSPs (high-scoring segment pairs) and interfering distances from three formulae (1, HSP length / total length; 2, identities / HSP length; 3, identities / total length) [54]. Table 7 shows the results of the pairwise comparisons between *L. methylohalidivorans* DSM 14336^T^ and the other seven genomes. As the results of the 16S rRNA analysis (Figure 1) revealed, the two *Leisingera* species *L. methylohalidivorans* and *L. aquimarina* show a close relationship, whereas *L. nanhaiensis* does not cluster together with the other two *Leisingera* species. The DDH similarities calculated *in silico* yielded similar results, indicating that the classification of *L. nanhaiensis* might need to be reconsidered. Furthermore, the DDH similarities of *L. methylohalidivorans* to *Phaeobacter* species are not significantly smaller, especially in the case of *P. caeruleus* and *P. daeponensis*, than to *L. aquimarina* and as already described to *L. nanhaiensis*.

**Table 7:** DDH similarities between *L. methylohalidivorans* DSM 14336^T^ and the other *Leisingera* and *Phaeobacter* species (with genome-sequenced type strains) calculated *in silico* with the GGDC server version 2.0 [55].

Reference species	HSP length / total length [%]	identities / HSP length [%]	identities / total length [%]
*L. aquimarina* (AXBE00000000)	52.40 ± 3.47	32.40 ± 2.46	47.00 ± 3.03
*L. nanhaiensis* (AXBG00000000)	14.50 ± 3.11	19.20 ± 2.29	14.60 ± 2.64
*P. arcticus* (AXBF00000000)	17.20 ± 3.28	20.40 ± 2.32	17.00 ± 2.77
*P. caeruleus* (AXBI00000000)	45.80 ± 3.41	27.00 ± 2.42	39.90 ± 3.01
*P. daeponensis* (AXBD00000000)	48.70 ± 3.43	26.90 ± 2.42	41.90 ± 3.01
*P. gallaeciensis* (AOQA01000000)	17.90 ± 3.31	21.00 ± 2.33	17.60 ± 2.80
*P. inhibens* (AXBB00000000)	18.50 ± 3.34	21.10 ± 2.33	18.10 ± 2.82

The standard deviations indicate the inherent uncertainty in estimating DDH values from intergenomic distances based on models derived from empirical test data sets (which are always limited in size); see [55] for details. The distance formulae are explained in [54]. The numbers in parentheses are GenBank accession numbers identifying the underlying genome sequences.
